# Effect of oriental medicine music therapy on patients with Hwa-byung: a study protocol for a randomized controlled trial

**DOI:** 10.1186/1745-6215-13-161

**Published:** 2012-09-11

**Authors:** Jeong-Su Park, Sunju Park, Chun-Hoo Cheon, Bo-Hyoung Jang, Song-Hee Lee, Seung-Hyun Lee, Sun-Yong Chung, Jong-Woo Kim, Chan-Yong Jeon, Jong-Hyeong Park, Yong-Cheol Shin, Seong-Gyu Ko

**Affiliations:** 1 Center for Clinical Research and Genomics, Korean Medical College, Kyung Hee University, Seoul, Republic of Korea; 2 Department of Preventive Medicine, Oriental Medical College, Kyung Hee University, Seoul, Republic of Korea; 3 Department of Oriental Medicine Music Therapy Center, Kyung Hee University Hospital at Gangdong, Seoul, Republic of Korea; 4 Hwa-byung Stress Clinic, Kyung Hee University Gangdong Oriental Medical Center, Seoul, Republic of Korea; 5 Department of Internal Medicine, College of Oriental Medicine, Gachon University, Seongnam, Republic of Korea

**Keywords:** Hwa-byung, Oriental medicine music therapy, Anxiety, Clinical trial

## Abstract

**Background:**

Hwa-byung, a Korean culture-bound syndrome with both psychological and somatic symptoms, is also known as ‘anger syndrome’. It includes various physical symptoms including anxiety, a feeling of overheating, a sensation of pressure on the chest, heart palpitations, respiratory stuffiness, insomnia, and anxiety.

**Methods/design:**

The proposed study is a single-center, double-blind, randomized, controlled trial with two parallel arms: an oriental medicine music therapy (OMMT) group and a control music therapy (CMT) group. In total, 48 patients will be enrolled into the trial. The first visit will be the screening visit. At baseline (visit 2), all participants fulfilling both the inclusion and the exclusion criteria will be split and randomly divided into two equal groups: the OMMT and the CMT (n = 24 each). Each group will receive treatment sessions over the course of 4 weeks, twice per week, for eight sessions in total. The primary outcome is the State-Trait Anxiety Inventory (STAI), and the secondary outcomes are the Hwa-byung scale (H-scale), the Center for Epidemiologic Studies Depression Scale (CES-D), the Hwa-byung visual analogue scale (H-VAS) for primary symptoms, the World Health Organization Quality of Life scale, brief version (WHOQOL-BREF), and levels of salivary cortisol. Patients will be asked to complete questionnaires at the baseline visit (visit 2), after the last treatment session (visit 9), and at 4 weeks after the end of all trial sessions (visit 10). From the baseline (visit 2) through the follow-up (visit 10), the entire process will take a total of 53 days.

**Discussion:**

This proposed study targets patients with Hwa-byung, especially those who have exhibited symptoms of anxiety. Therefore, the primary outcome is set to measure the level of anxiety. OMMT is music therapy combined with traditional Korean medicinal theories. Unlike previously reported music therapies, for which patients simply listen to music passively, in OMMT, patients actively move their bodies and play the music. Because Hwa-byung is caused by an accumulation of blocked emotions and anger inside the body, OMMT, because of its active component, is expected to be more efficacious than pre-existing music therapies.

**Trial registration:**

Current Controlled Trials ISRCTN11939282

## Background

Hwa-byung is a syndrome caused by unrelieved anger accumulated inside the body. As anger builds up in the patient and fails to be released, the patient reaches a breaking point and explodes in anger and rage. These emotions are categorized as ‘fire’ in Korean medicine, hence the name, ‘fire sickness’. In th*e Diagnostic and Statistical Manual of Mental Disorders*, Fourth Edition (DSM-IV) and in the USA, Hwa-byung is named ‘anger syndrome’ and though to be a Korean culture-bound illness. The annual occurrence of Hwa-byung is approximately 4.2 to 11.9%, and often occurs in middle-aged patients. Because the illness is usually caused by stress occurring over a relatively long period, and symptoms continue even in the absence of the factors that caused the unresolved symptoms, many patients think they are unlikely to be cured.

Patients with Hwa-byung are often diagnosed with major depression, anxiety disorders, or somatization disorders. According to a study by Kim [1], the probability of patients having both Hwa-byung and major depression was 26.9%, and the patient group diagnosed with Hwa-byung based on the Hwa-byung Structured Clinical Interview for DSM-IV (SCID) had significantly increased evaluation indexes of the State-Trait Anxiety Inventory (STAI) the Center for Epidemiologic Studies Depression Scale (CES-D), and the Hwa-byung scale (H-scale) than patients not diagnosed with Hwa-byung. Music therapy has been reported to relax, sedate, and improve vital signs in patients [2], and is effective when the patient is undergoing, about to start, or just ending invasive medical examinations or procedures [3,4]. In addition, music therapy has been reported to reduce the level of salivary cortisol [5]. In Korea, cases have been reported where the symptoms of children with attention deficit hyperactivity disorder were reduced after receiving oriental medicine music therapy (OMMT) [6], and students with hearing impairment experienced less stress after playing traditional musical instruments [7].

OMMT is based on the idea that each oriental musical factor contains different energy, or *qi*. Music with different types of *qi* has different qualities that affect the mind and body of the patients. OMMT aims to control *qi* through stimulation by music, and eventually to achieve balance in both the mind and body [8].

Until recently, clinical study of OMMT was resticted mainly to patients with cerebral infarction and cancer of the blood. Classifying patients in accordance with Sasang body constitutions (four types) and applying OMMT to patients based on their body constitution was reported to significantly improve blood flow to the brain [9]. Cases were reported in which hemiplegia and walking impairment due to cerebral infarction improved after OMMT [10]. It has been reported that leukocyte numbers and absolute neutrophil count (ANC) improved in patients with blood cancer after treatment with OMMT [11]. There have been previous studies on the music therapy for patients with Hwa-byung, but to our knowledge, there has been no clinical investigations into the effects of OMMT on Hwa-byung.

Hwa-byung is linked with emotions such as fury. When a person is unable to release such emotions and reached a certain level of frustration, these emotions accumulate inside the body, leading to the person having an outburst of temper (considered to be a form that resembles fire). OMMT is an active movement therapy that includes movement of the body and instruments. Therefore, treatment with OMMT is expected to produce positive effects for patients with Hwa-byung. None of the previous studies on music therapy for Hwa-byung has included the physical component of movement along with the music.

## Methods/design

### Hypothesis

Our hypothesis is that OMMT will produce superior reduction in the STAI measurements compared with a control music therapy that has no physical component.

### Objectives

The aim of this proposed study is to determine the effectiveness of OMMT compared with the control music therapy (CMT) in Hwa-byung patients, especially the anxiety. The aims are: 1) to produce a clinically significant improvement in the STAI scores of patients with Hwa-byung patients; 2) to measure the effect of OMMT on the levels of salivary cortisol; and 2) to identify changes in scores on the H-scale, the CES-D, the Hwa-byung visual analogue scale (H-VAS) for primary symptoms, and the World Health Organization Quality of Life scale, brief version (WHOQOL-BREF) after OMMT

### Setting

**Figure 1:**
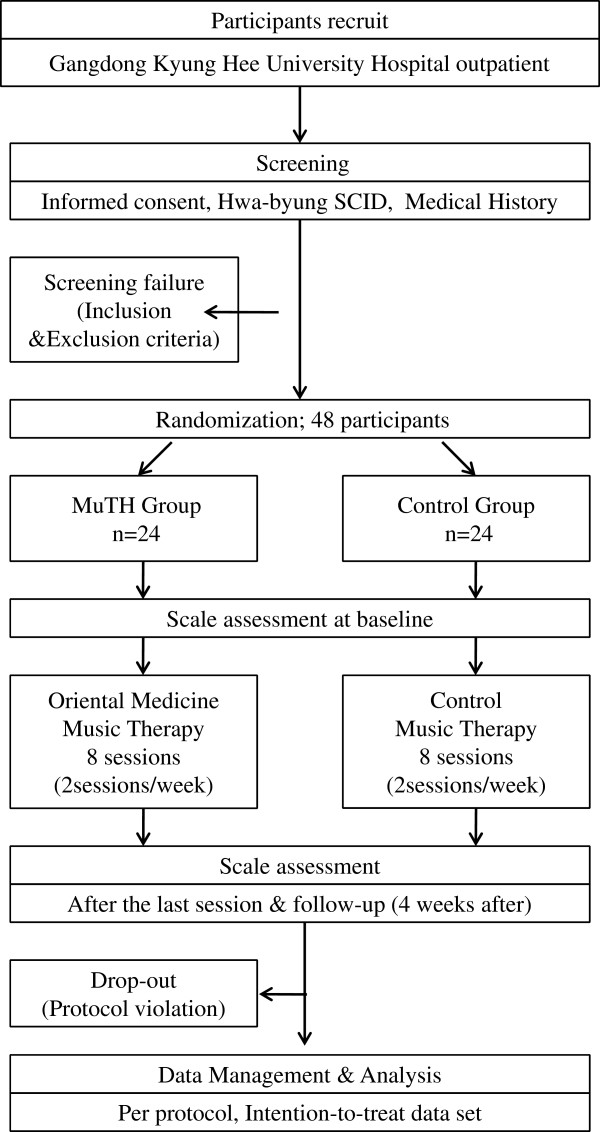
Study flow chart.

**Figure 2:**
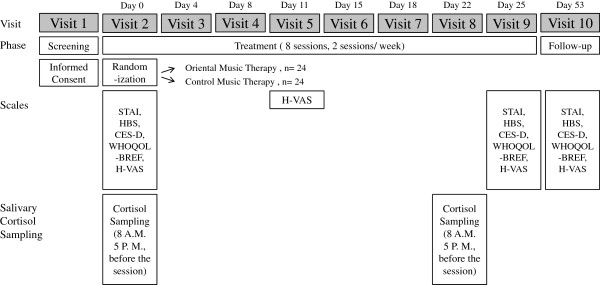
Scales assessment schedule.

This trial is a single-center, double-blinded, parallel-group randomized controlled trial, which will be performed at the Hwa-byung Stress Clinic, Kyung Hee University Hospital in Gangdong, Seoul. Patients will be recruited through internet advertisements, newspaper advertisements, and official notices posted in the hospital. Patients recruited for the trial will be required to visit the investigational site and sign a written informed consent form. Patients who sign the consent form, who are diagnosed as having Hwa-byung by the Hwa-byung Structured Clinical Interview for DSM (SCID), and who satisfy the inclusion and exclusion criteria will be registered into the clinical trial. The registered patients will be asked to complete the STAI, the H-scale, the CES-D, the H-VAS, and the WHOQOL-BREF. Once 16 patients have been registered randomization will commence, with equal numbers allotted to the the OMMT group and the CMT group. This randomization process will be repeated three times, until 48 patients are divided equally between the two groups. For 4 weeks the patients will receive either OMMT or CMT in groups of eight patients. Patients will complete the H-VAS at sessions 1, 4 and 8 of the trial, and also after session 8 (in week 4). Salivary cortisol will be collected on the day before session 1, and at session 8., and will be collected twice, at 0800 and again at 1700 hours. The time between the screening tests to the end of the trial may vary depending on the patient schedule. From the first session to the last follow-up in week 8, it will take approximately 53 days to complete the study. Figure 1 is an overview of this proposed trial, and Figure 2 is an approximate visit schedule.

### Inclusion criteria

The inclusion criteria are as follows.

• Male or female over the age of 20 years.

• Diagnosed as having Hwa-byung using the Hwa-byung SCID for DSM-IV.

• Provided written informed consent.

• Have no problems with communication (for example, reading, writing, listening, speaking).

### Exclusion criteria

The exclusion criteria of this trial are as follows.

• In need of regular medication or psychotherapy.

• Has a severe neurological or psychiatric disorder.

• Has a history of major neuropsychiatric disorder (for example, autism, learning disorder, mental retardation).

• Has had a change in medication of anti-depressants or barbiturates in the past 1 month.

• Is seriously irritable.

• Participated in any other clinical trials in the past one month from the screening day.

• Had a regular mind/body relaxation training in the past 1 year, such as music therapy, qigong, yoga, or meditation.

• Cannot understand written informed consent form or follow this study.

• Presence of mental retardation and mental or emotional problems.

### Assessments

Various assessments will be carried out at specific visits (see Table 1).

**Table 1:** Summary of assessments at procedures (x shows the items to be carried out at each visit)

**Procedure**	**Screening (visit 1)**^**1**^	**Treatment phase**								**Follow-up (visit 10)**
		**Visit**								
		**2**^**2**^	**3**	**4**^**3**^	**5**	**6**	**7**	**8**	**9**	
Obtain informed consent	x									
Hwa-byung SCID	x									
Take history^1^	x									
Collect demographic information^2^	x									
Randomization		x								
Scales										
STAI		x							x	
H-scale		x							x	
CES-D		x							x	
WHOQOL-BREF		x							x	
H-VAS		x			x				x	x
Measure salivary cortisol		x							x	
Treatment session		x	x	x	x	x	x	x	x	
Safety assessment^9^			x	x	x	x	x	x	x	x

Abbreviations: CES-D, Center for Epidemiologic Studies Depression Scale; H-scale, Hwa-byung scale; H-VAS, Hwa-byung visual analogue scale for primary symptoms; STAI, State-Trait Anxiety Inventory (STAI); WHOQOL-BREF, World Health Organization Quality of Life scale, brief version.

^1^ Visit 1: acquisition of informed consent and performance of screening procedure.

^2^ Visit 2: randomization and performance of outcome assessment for baseline.

^3^ Visit 4: performance of outcome assessment for primary objective.

4 Drug history for the past month, allergy and disease history, obstetric history, history of menarche and menstrual periods.

^5^ Date of birth, age, height, and weight.

^6^ Medical examination by interview.

^7^ A scale for diagnosing blood stagnation; has not been published.

^8^ 100 mm visual analogue scale to measure mean and the maximum pain during the menstrual period.

^9^ Measurement of aspartate and alanine aminotransferases, blood urea nitrogen, creatinine, and alkaline phosphatase.

^10^ White and red blood cell counts, hemoglobin, hematocrit, platelets.

^11^ Protein, glucose, urobilinogen, ketone, red and white blood cells, squamous cells.

^12^ Medical examination and self-report of adverse events.

### Interventions

#### Oriental medicine music therapy

The OMMT program entails eight sessions of 60 minutes each, conducted twice a week over a period of 4 weeks, and composed of five different levels of activity.

The first level is abdominal breathing for 5 minutes; patients breathe rhythmically to slow Korean folk rhythms called *Jinyangjo* played by *Daegum*, a kind of traditional Korean flute. The purpose during this portion of the session is to begin breathing control before the start of the therapy itself. Daegum has the quality of decreasing qi.

The second level involves vocalization and sound. Patients are asked to make the sounds of *Kung*, *Duk*, and *Dung*, which are traditional Korean methods of pronouncing rhythm, using their own voice. This portion of the therapy is conducted twice a week for four weeks in a total of eight sessions 10 minutes each.

The third level of treatment is the five-element rhythm therapy, which takes 10 minutes. Patients stimulate five acupuncture points (GB-20, GB-21, PC-6, ST-40, and GB-31) with maracas to the rhythm of music that has the Wood-qi element of the five elements of music. The objective of this portion is to circulate the blood and *qi* using Wood-*qi* music, which has active characteristics when stimulated.

The fourth level is 10 minutes of spreading music therapy, which lets patients play music themselves using *Janggu*, a traditional percussion instrument.

The fifth level is a 10-minute session of relieving music therapy, in which patients beat out a rhythm with wood blocks, which induces Wood-*qi*. This is performed to the rhythm of Wood-*qi* music, which stimulates the liver *qi*.

The last level is singing with abdominal breathing for 15 minutes. Patients make the sounds of ‘mo’ and ‘mi’, out of ‘ma’, ‘me’, ‘mi’, ‘mo’ and ‘mu’, which have *yin* characteristics

#### Control music therapy

The control music therapy program is also carried out for 60 minutes twice a week for 4 weeks, to give a total of eight sessions. Sessions 1 to 4 involve the patients playing different types of traditional music. Each session consists of three or four different types of music and each has the characteristic of reducing *qi*, making people calm, and lightly stimulating the mind from calmness, heartbreaking sadness, and happiness. From session 1 to 4, the music will be different at each session. The music played in sessions 5 to 8 will be the same music played in the session 1 to 4, but the music will be different at each session.

### Outcomes

#### Primary outcome

The primary outcome of this study is the change in the STAI between visit 2 (before treatment and visit 9 (after all sessions are completed).

#### Secondary outcome

The secondary outcome measures are the level of salivary cortisol and the scores on the H-scale, the CES-D, the H-VAS, and the WHOQOL-BREF.

### Safety assessment

All participants will report any adverse events (AEs) experienced during the treatment or follow-up phases. Every AE will be recorded on the case report form (CRF). If the AE is severe and associated with the trial, the participant will be withdrawn from the trial and given medical treatment.

### Sample size

The primary outcome in this trial is the change in STAI between the baseline (visit 2) and after treatment completion (visit 9). The hypothesis is: H0: δ = Δ1-Δ2 < 0 and H1: δ = Δ1-Δ2 ≥ 0, where Δ1 is change in STAI between baseline and visit 9 and the baseline in the OMMT group, and Δ2: is change in STAI between baseline and visit 9 in the control group.

The sample size was calculated based on a *qigong* study of heart disease [12]. In this study, the participants were divided into two groups: the ‘*qigong*’ and ‘relaxing’ groups. After eight sessions, in the ‘*qigong*’ group, the mean change in STAI-1 was 8.82 and the standard deviation was 10.385, which is in accordance with a two-tailed, α error of 5% and power of 80% shown in the following equation.

(1) $$N=\frac{2{\left(1.96+0.84\right)}^{2}10.385^{2}}{=22}$$

Therefore, we will be randomly assigning 24 participants to each group, assuming a 10% drop-out rate.

### Randomization

Participants will be randomized during visit 2. The randomization process will be started after 16 patients who passed the screening test are enrolled, and randomization will be carried out three times in total. The steps of the randomization will be overseen by an institution unrelated to this trial. Except for the therapist playing the music, anyone related to the trial (investigators, participants, and monitors) will be blinded to the type of music therapy being used, and the outcomes will be evaluated by the investigators, not the therapist. The first step of the randomization process will be to generate a random number to allocate the patient (SPSS software, SPSS Inc., Chicago, IL, USA). The institution in charge of randomization will label the patients as ‘A’ or ‘B’ and report to the trial center, who will randomize the patients accordingly and assign them to the following sessions. The ratio of the OMMT group and the CMT group is 1:1.

### Statistical method

The primary endpoint is the STAI. Efficacy analysis will be performed both as per protocol (PP; participants completed the trial without any protocol violation) and as intention to treat (ITT; all randomly assigned participants). The PP dataset will comprise all the patients who completed all the treatment sessions and questionnaires, while the ITT dataset will comprise patients who received at least one treatment session.

The independent *t*-test will be used to compare the changes in the STAI between visit 2 (baseline) and visit 9 between the OMMT group and the control group. Analysis of covariance (ANCOVA), using the baseline score as a covariate, will be used to analyze the mean change in the STAI, the H-scale, the CES-D, the H-VAS, and the WHOQOL-BREF, and in salivary cortisol levels between the two groups.

Other issues not included in this protocol will follow the Korean Food and Drug Agency Clinical Statistical Guidelines (Medicine 65625–13353, 2000. 12.29).

### Monitoring

The Center for Clinical Research and Genomics (CCRG; the contract research organization) will monitor this study. The monitoring procedures will follow the Standard Operating Procedure (SOP)s. Monitoring will begin after the first participant completes the whole period of this study.

### Ethical considerations

The institutional review board (IRB) has approved this clinical trial before participant recruitment. The reference number is KHNMC-OH-IRB 2010–014 (IRB of Kyung Hee University Gangdong Medical Center, approved on 1^st^ of April 2011). Before the trial, the protocol, the CRF, and all the documents to be reviewed by the patients have been previously approved by the IRB of Kyung Hee University Hospital. The trial will be conducted in accordance with the Declaration of Helsinki 2008 and/or the principles of good clinical practice of the Korean Food and Drugs Administration. Before undertaking any study-related procedures, all participants will provide written informed consent.

## Discussion

There is no gold standard of measurement for Hwa-byung. However, anxiety is one of the main symptoms of Hwa-byung, and is a symptom with measurable endpoints, which enables doctors to quantify the level. This is why the application of the STAI to measure the level of Hwa-byung was chosen as the primary endpoint.

This study will use listening to traditional music as the therapy for the control group. Traditional music therapy is not specifically a placebo therapy, as it has been reported that traditional music therapy shows effects for a diverse number of psychological disorders. However, there is no doubt that OMMT is different from the listening to traditional music. The OMMT is based on traditional Korean medicinal theories such as the *yin*/*yang* theory and meridian theory. OMMT does not involve the passive listening to music; patients actively move their body, play the instruments, and thereby release stress. Although similar elements of music will be used, the essence of the two therapies is very different.

## Trial status

This clinical trial is currently recruiting participants.

## Abbreviations

CES-D: The Center for Epidemiologic Studies Depression Scale; CRF: Case Report Form; DSM-IV: Diagnostic and Statistical Manual of Mental Disorders, Fourth Edition; H-scale: Hwa-byung scale; H-VAS: Hwa-byung primary symptoms Visual Analogue Scale; OMMT: Oriental medicine music therapy; SCID: Structured Clinical Interview for DSM-IV; STAI: State-Trait Anxiety Inventory; WHOQOL: World Health Organization Quality of Life.

## Competing interests

The authors declare that they have no competing interests.

## Authors’ contributions

JSP, SJP, and CHC have written the first draft manuscript for this trial and calculated the sample size. They will also contribute to the monitoring of this trial. SYC and JWK are the site investigators, and they began and directed this study. SHL wrote the section on OMMT and the control music therapy. SGK has conducted all the procedures for this protocol. BHJ, SHL, CYJ, JHP, YCS and SGK have edited the first manuscript. All authors read and approved the final manuscript.
